# The BSR Celebrates Researchers in Academic Radiology

**DOI:** 10.5334/jbr-btr.1455

**Published:** 2017-11-18

**Authors:** Alain Nchimi

**Affiliations:** 1 University of Liège and Université Libre de Bruxelles, BE

**Keywords:** Research, Academic, Radiology, Thesis

Research in academic radiology is the reservoir for future knowledge and education that are cornerstones of the radiology future. Consequently, promoting, encouraging and bringing to light research in academic radiology have always been major goals of the Belgian Society of Radiology. This year’s symposium makes no exception to that rule and emphasizes that the Belgian radiology has a bright future and still produce high profile academic researchers.

In this article, the selected researchers, all recent PhDs funded by the BSR, present themselves and summarize their long-standing work and academic careers. The summaries of their doctoral thesis are available on the site of the Journal of the Belgian Society of radiology https://www.jbsr.be/.

**Figure:**
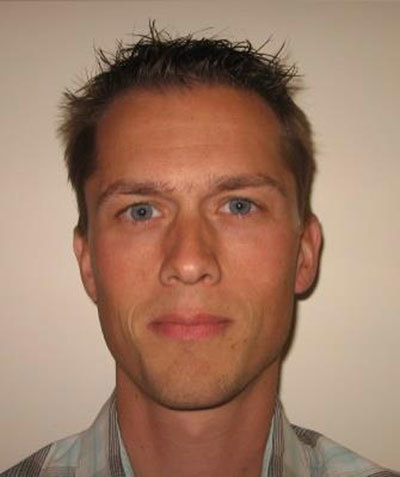
Pieter Julien Luc De Visschere (2016)

Pieter De Visschere graduated in medicine at Ghent University in 2004 and became a radiologist at the same university in 2009. Dr. De Visschere has been a staff member at the department of Radiology at Ghent University Hospital since 2009, and has a special interest in genitourinary radiology and mammography. Since 2013, he has also been radiologist-second/third reader in the Flemish breast cancer screening program.

He is the author and co-author of about 30 publications in national and international journals, 16 of which as first author. He obtained his PhD in December 2016 with a thesis entitled ‘Improving the diagnosis of clinically significant prostate cancer with magnetic resonance imaging’ with promotor Prof. Dr. Geert Villeirs. Dr. De Visschere presented about 50 lectures on genitourinary and mammographic topics during national and international meetings. He is a member of the Belgian Society of Radiology (BSR), European Society of Radiology (ESR), European Society of Urogenital Radiology (ESUR), European Association of Urology (EAU) and the Young Academic Urologists (YAU) Prostate Cancer Working Party. He is member of the genitourinary scientific subcommittee of the European Congress of Radiology (ECR) 2016, 2017 and 2019.

His thesis main teaching points are:

1. The prostate Magnetic Resonance Imaging (MRI) protocol has been changing over the years. With the currently available techniques, the highest image quality and efficiency seems to be obtained with a short scan protocol consisting of only T2-Weighted and Diffusion-Weighted Images. This offers sufficient information and accuracy for most diagnostic indications. Only in doubtful or complicated cases, Dynamic Contrast Enhanced Imaging or Magnetic Resonance Spectroscopic Imaging may be performed additionally.
2. The accuracy of prostate MRI to detect clinically significant prostate cancer varies with the applied definition of clinically significant disease. The major value of prostate MRI is to demonstrate or exclude high-grade and large tumours. Prostate MRI offers additional information, next to clinical biomarkers such as PSA level and digital rectal examination, to determine the likelihood of a clinically significant PC. Prostate MRI may improve the diagnosis of clinically significant PC by serving as an additional decision tool to triage patients with elevated PSA towards immediate biopsy or not.

**Figure:**
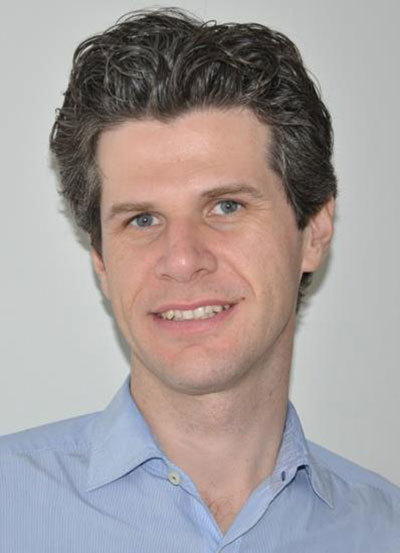
Olivier Ghekiere (2017)

Dr. Olivier Ghekiere currently works as radiologist at Jessa Hospital in Hasselt and at CHC Hospital in Liège. His main field of interest is cardiac imaging. He graduated in Radiology in 2006 at the University Hospital Saint-Luc in Brussels (Prof. Bruno Vande Berg). He is an active member of the executive board of the Belgian Society of Radiology (BSR), the European Society of Cardiac Radiology (ESCR) and BELMIP (Belgian Medical Imaging Platform) at the Federal Public Service Social Security.

Dr. Ghekiere initiated research in 2010 which resulted in a doctoral thesis proposal at the Hasselt University in January 2014. He obtained his PhD degree in September 2017 at the Hasselt University, for a dissertation entitled ‘Contribution of cross-sectional imaging in the work-up of intermediate coronary artery stenosis’, which was supported by a grant of the Belgian Society of Radiology. The results of this research were already presented at the RSNA meetings in Chicago in 2011, 2014 and 2017, at the European Congress of Radiology (ECR) congress in Vienna in 2016, at the Journées Françaises de Radiologie (JFR) in Paris in 2012, and at the European Society of Cardiac Radiology (ESCR), annual scientific meetings in 2012 in Barcelona, 2014 in Paris and 2017 in Milan. He was awarded Magna cum laude for his scientific work “Technical Pitfalls in cardiac CT angiography: What the radiologist should know” at the ESCR annual scientific meeting 2012 in Barcelona.

The goal of his doctoral thesis was to investigate how recent advances in non-invasive cardiac imaging, including quantitative coronary Computed Tomography Angiography (CTA), non-invasive Fractional Flow Reserve (FFR) estimates from CT (FFR_CT_) and stress perfusion cardiac magnetic resonance (CMR) can represent alternatives in the management of intermediate coronary artery stenosis (i.e. 40–70% diameter reduction). In summary, angiographic anatomical parameters using quantitative coronary CTA and coronary angiography poorly predict the functional significance of intermediate-grade stenosis. Even with the most recent technological advances in coronary CTA, additional functional evaluation is required in this range of stenosis to guide the therapeutic management. Functional assessment of intermediate-grade coronary stenosis by both stress perfusion CMR and FFR_CT_ allows strong correlation with invasive FFR measurement. Both techniques may be used as a safe non-invasive gatekeeper to guide management of intermediate-grade stenosis. Assessment of the functional significance of an intermediate coronary stenosis estimated from stress perfusion CMR should be corrected for perfusion changes in remote myocardium to improve both the correlation with catheter FFR and the diagnostic accuracy to predict significant (ie: FFR ≤ 0.80) lesions.

**Figure:**
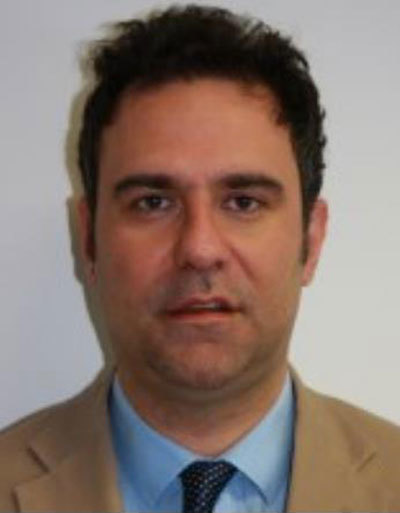
Paolo Simoni (2015)

Prof. Paolo Simoni earned his medical degree at the University of Rome “La Sapienza” in 2002 and completed his residency in medical imaging in Rome in 2006 at University Hospital Campus-Bio-Medico of Rome). Prof. Paolo Simoni also got a Master in Business and Administration at University Paris 1 Panthéon-Sorbonne (2017).

From 2007 to 2010, he attended an internship in Musculoskeletal Imaging at Cliniques Universitaires Saint-Luc in Brussels (UCL). He was therefore appointed Head of the Musculoskeletal service of the University Hospital of Liège Form 2010 to March 2015.

From June 2015 he heads of the Department of Paediatric Imaging et Children’s University Hospital “Queen Fabiola” in Brussels, with a special interest in the Paediatric Musculoskeletal Imaging. He was appointed Professor of Paediatric Imaging at Université Libre de Bruxelles (ULB) in 2016. He is currently a permanent invited member of the Board of the Belgian Society of Radiology (BSR) and elected member of the sub-committee for Arthritis of the European Society of Skeletal Radiology (ESSR). He took a PhD in Medical Science from the University of Liège in March 2015 for an original work entitled “Optimisation of X-rays imaging techniques for the assessment of the joint space”.

The aim of his work was the optimisation of X-ray-based medical imaging techniques in different clinical and experimental settings. X-ray-based medical imaging techniques are still the most widely used techniques for imaging the degenerative and inflammatory joint diseases. In addition, these techniques allow a direct visualisation of the subcondral bone and the subchondral bone plate, essential components of the bone-cartilage unit that cannot be visualised on MRI because of the lack of signal.

The first article of the thesis was carried out to assess the accuracy and the reproducibility of X-rays in evaluating the joint space measurement compared to quantitative MRI; the patello-femoral joint was studied as a paradigm because its inherent anatomical and biomechanical complexity. The second article was an experimental study performed in cadavers to optimise the visibility of the cartilage and the subchondral bone with a significant decrease of the radiation burden. The radiation burden reduction in this ex vivo and in vitro study without compromise in the image quality allows a significant of optimisation of computed tomography arthrography of the hip in the routine clinical practice. The last article devoted to advances in tomosynthesis, a recent X-ray technique performing a set of low-dose tomographic images. The accuracy of tomosynthesis to detect the joint erosions of the forefoot in patients with proven rheumatoid arthritis was compared to that of standard X-rays using computed tomography as a gold standard. This work suggested a promising role of tomosynthesis in the follow-up of the joint erosion of the forefoot. In summary, X-ray-based medical imaging techniques remain valuable and evolving tools to evaluate the joint space and the mineral components, which can be complementary or alternative to MRI.

**Figure:**
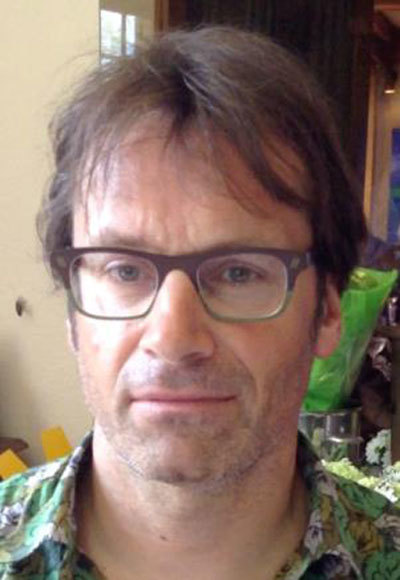
Peter Vanlangenhove (2016)

Dr. Vanlangenhove was certified as a candidate in Medical Science at the University of Ghent in 1987. In 1991 he graduated as a MD in Medicine, Surgery and Obstetrics at the University of Ghent. From 1991 until 1996 he was a resident at the Department of Radiology at the University hospital of Ghent under the supervision of Professor E. Vandevelde and Professor M. Kunnen. In July 1996 he earned the certificate of Radioprotection and Radiation Physics.

From that moment on he has been interested in the therapeutic part of radiology. In the period between 1996 to1999 he was a part-time fellow at the Interventional and Diagnostic Radiology Department at the University Hospital of Ghent (under the supervision of Professor. L. Defreyne) and part-time fellow at the Department of Vascular Ultrasound (under the supervision of Professor. D. Voet). In 1999 he became Adjunct-chief of clinics at the Department of Interventional and Diagnostic Radiology. He limited his work at the Department of Vascular Ultrasound to one day a week performing the mapping of venous insufficiency of the lower legs. He worked in the Department of Vascular and Interventional Radiology (VINRAD) with special interest in venous interventions. During this period he started to work on his PhD.

In 2011 he was granted a Clinical Investigation Fund at the University hospital of Ghent which resulted in April 2015 in a PhD degree; “Contributions to the pathophysiology and treatment of varicoceles”. The first part of this work demonstrated that the endovascular treatment of varicoceles with glue is an efficient, safe and tolerable method. The second part of this work, delivered contributions to the research of the pathophysiology of varicoceles and its association with infertility. He published several A1-publications regarding this topic.

Dr. Vanlangenhove is currently Chief of Clinics at the Interventional and Diagnostic Radiology Department at the University Hospital of Ghent.

**Figure:**
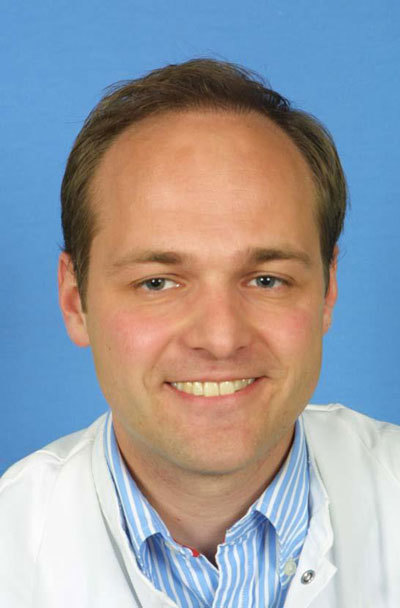
Tom Verstraeten (2014)

Dr. Verstraeten earned his MD and Master in Sports Medicine in 2011 at the Ghent University. He started as a resident Orthopedic Surgery and Traumatology at the AZ Sint-Jan Hospital Brugge (Dr. Bruno Vandekerckhove) during 2011–2013. Subsequently, he was a resident radiology at the AZ Sint-Jan Hospital Brugge (Prof Jan Casselman) in 2013–2014 and at the University Hospital Ghent in 2014–2017. During his medical degree training he was also partly responsible for the surgical prelevation of the meniscal transplants (Prof. Verdonk).

In 2015, he was selected by the European Society of Radiology (ESOR) to be a visiting fellow at the University of Heidelberg, Germany. In the department of Radiology and Orthopedic Surgery he was trained in Musculoskeletal Radiology, mentored by Prof. Dr. Med. M.A. Weber. In 2017, he was certified with the European Diploma of Radiology (EDiR).

During his clinical orthopedic work, Dr. Verstraeten also worked on his PhD research project where he investigated the 3D anatomy of the glenoid and the proximal humerus, mentored by Prof Lieven De Wilde and Prof Jan Victor of the Orthopedic Surgery and Traumatology department at the University Hospital Ghent. He developed a new manner to describe and quantify the gleno-humeral relationship and the surgical planes of the shoulder joint based on 3D CT-scan images. Due to these new insights in the 3D anatomy of the shoulder joint, he was able to contribute in the development of a new surgical device. This new surgical device makes it possible to position the glenoid component in an anatomic total shoulder prosthesis in a more accurate way during surgery. By describing the normal 3D anatomy of the glenoid using 3D CT-scan imaging, new research can be done to understand and treat pathology of the shoulder.

He published several A1-publications regarding this topic and earned his PhD-degree in 2014. In 2015, this work was presented at the European congress of Radiology and in 2016, he received an award during the Musculoskeletal section meeting of the Belgian Society of Radiology.

Dr. Verstraeten is currently a fellow at the Onze Lieve Vrouw (OLV) Hospital Aalst, Belgium.

